# Studies of the Renal Circulation
*From the Nuffield Institute for Medical Research, Oxford. Pp. xiii 187. 83 Illustrations. Price 25/-. Oxford: Blackwell Scientific Publications, 1947.


**Published:** 1948

**Authors:** Josep Trueta, A. E. Barclay, K. J. Franklin, Marjorie M. L. Prichard


### STUDIES OF THE RENAL CIRCULATION.*

BY

JOSEP TRUETA, M.D., A. E. BARCLAY, D.M., K. J. FRANKLIN, D.M. and MARJORIE M. L. PRICHARD, M.A.

### A REVIEW

IN 1942 Professor Josep Trueta discovered that prolonged constriction of a limb could cause persistent spasm of its main arteries ; and not only of the main arteries of the constricted limb but also of arteries proximal to the level of the constriction. Furthermore, it could cause a reflex spasm of the arteries of the opposite limb (thigh). He was at that time investigating the " crush syndrome " and its resultant anuria. Continuing his researches, he studied the effect of constriction of a lower limb upon the abdominal and in particular the renal vessels. In order to ensure meticulous accuracy he gathered together a team, consisting of Dr. A. E. Barclay, radiologist, Dr. K. J. Franklin, physiologist, Miss M. N. L. Prichard and Dr. P. M. Daniel, microscopic anatomist. With this team the physiological data collected from radiological studies were integrated with visual observations on the circulation of the kidney and with the relevant anatomical findings. A technique of angiography was developed in which by injection of thorotrast into the jugular vein of rabbits the abdominal vessels could be radiographically demonstrated without opening the abdomen. By cineradiography the time taken by the contrast medium to reach the renal artery and vein respectively was determined. The calibre of the vessels could also be measured. Having established the technique, a series of tourniquet experiments was made. These revealed that a remarkable constriction of the renal artery might occur in anaesthetized rabbits after a tourniquet had been applied to one thigh. Further experiments showed a similar constriction of the renal artery after stimulation of the central end of a divided sciatic nerve ; or in an even more marked degree after splanchnic nerve stimulation. An unexpected observation was made in the course of these experiments, namely that after the application of a tourniquet the intra-renal circuit-time might be noticeably reduced. This gave rise to a suspicion that a short-circuiting of the blood flow might occur in the rabbit's kidney.

Next, the intra-renal vascular pattern was studied in order to discover the actual pathway used when this short-circuiting occurred. From injection experiments it appeared that :—

1. In the normal animal the cortical vessels receive the greater

*From the Nuffield Institute for Medical Research, Oxford. Pp. xiii 187. 83 Illustrations. Price 25/-. Oxford : Blackwell Scientific Publications, 1947.

part of the renal blood supply, whilst those of the medulla receive a noticeably smaller part;

2. In the stimulated animal the vessels of the medulla receive by far the greater part of the blood supply; whilst those of the cortex, excepting the vessels in its deepest zone, receive virtually none;

3. The vessels of the medulla, carrying in the normal animal a relatively small part and in the stimulated animal the bulk of the total intra-renal blood-flow, were apparently the same in each case, namely, the vasa recta.

From the examination of injected kidneys it was seen that the glomeruli of the kidneys are of two kinds, varying with their position in the cortex. The glomeruli in the deepest zone of the cortex are larger than the glomeruli which lie nearer the periphery. Moreover, the efferent arteriole from the peripheral glomerulus is smaller than the afferent arteriole to the same glomerulus: whereas the efferent arteriole from the deeper-seated (or juxta-medullary) glomerulus is often nearly as large as the afferent arteriole to that glomerulus. These juxta-medullary glomeruli, lying in the deepest zone of the cortex, are distinguished by an efferent vessel of large calibre, which passes into the medulla to divide into the vasa recta. The cortical glomeruli have an efferent vessel of small calibre which breaks up to form part of the cortical intra-tubular capillary network. Some of these cortical glomeruli lie in all zones of the cortex, including the deepest or juxta-medullary zone. So the difference is one of structure not merely of situation.

Thus Trueta and his co-workers conclude that the vasa recta provide a pathway of considerable capacity, which makes it possible for the blood to pass from the arcuate and proximal paths of the interlobular arteries, via the juxta-medullary glomeruli, to the corresponding veins without traversing a capillary network. They have demonstrated that the kidney has two potential circulations. Blood may pass either almost exclusively through one or other of two pathways, or to a varying degree through both. The circulation associated with the cortical glomeruli is the greater circulation: that associated with the juxta-medullary glomeruli is the lesser.

Another significant difference was noted by Peter (in 1909)—that there are two types of nephron: one with a long and the other with a short loop of Henle. The tubule with the long loop of Henle arises from a juxta-medullary glomerulus, situated in the deepest part of the cortex: and that with the short loop arises from a cortical glomerulus lying in the superficial part of the cortex. The most striking feature in the different tubules is that the greater part of the difference in their length lies in variations in the length of the thin segment of the loop of Henle. This segment is longest in tubules arising from juxta-medullary glomeruli. The vessels which constitute the medullary by-pass are those associated with the juxta-medullary glomeruli.

There was ample evidence that the circulation through the cortex could be either diminished, or even, in some circumstances, totally arrested, while a circulation continued through the medulla. In ordinary circumstances the greater part of the blood circulates through the cortex and the majority of the important functional elements of the kidney are situated there : so that any diversion of the circulating blood away from this part of the kidney is of profound significance.

We cannot, in this review, follow out the various conjectures and speculations upon which the authors venture. It is to be hoped that their researches will continue. So far, what has been ascertained regarding the dual circulation within the kidney is of vital importance in clinical medicine.

Rightly may Professor Trueta say that it has been of great good fortune that the Nuffield Institute for Medical Research, Oxford, holds the door open to clinicians with problems to solve. We cannot praise too highly the illustrations in this volume, nor the pains taking work that made the experiments and the specimens resulting from them possible. One is tempted to think that this discovery and explanation of the dual circulation in the kidney is one that will rank with Bell's discovery of the functions of the anterior and posterior spinal nerve roots.

